# Integrated Analysis of Physiological Responses and Transcriptome of Cotton Seedlings Under Drought Stress

**DOI:** 10.3390/ijms26167824

**Published:** 2025-08-13

**Authors:** Xin Li, Yuhao Zhao, Chen Gao, Xiaoya Li, Kunkun Wu, Meiwei Lin, Weihong Sun

**Affiliations:** School of Agricultural Engineering, Jiangsu University, 301 Xuefu Road, Zhenjiang 212013, China; 2212316010@stmail.ujs.edu.cn (X.L.); 2212316004@stmail.ujs.edu.cn (Y.Z.); 2212216007@stmail.ujs.edu.cn (C.G.); 2212416005@stmail.ujs.edu.cn (X.L.); 2212416004@stmail.ujs.edu.cn (K.W.); 2212216002@stmail.ujs.edu.cn (M.L.)

**Keywords:** drought stress, cotton seedings, gene expression, transcriptome analysis

## Abstract

Investigating the physiological responses and resistance mechanisms in plants under drought stress provides critical insights for optimizing irrigation water utilization efficiency and promoting the development of irrigation science. In this study, cotton seedlings were cultivated in a light incubator. Three drought stress levels were applied: mild (M1, 50–55% field moisture), moderate (M2, 45–50%), and severe (M3, 40–45%). Transcriptome analysis was performed under mild and severe stress. The results revealed that differentially expressed genes (DEGs) related to proline degradation were down-regulated and proline content increased in cotton. Under different stress treatments, cotton exhibited a stress-intensity-dependent regulation of carbohydrate metabolism and soluble sugar content decreased and then increased. And the malondialdehyde content analysis revealed a dose-dependent relationship between stress intensity and membrane lipid peroxidation. Stress activated the antioxidant system, leading to the down-regulation of DEGs for reactive oxygen species production in the mitogen-activated protein kinase (MAPK) signaling pathway. Concurrently, superoxide dismutase activity and peroxidase content increased to mitigate oxidative damage. Meanwhile, the photosynthetic performance of cotton seedlings was inhibited. Chlorophyll content, stomatal conductance, the net photosynthetic rate, the transpiration rate and water use efficiency were significantly reduced; intercellular carbon dioxide concentration and leaf stomatal limitation value increased. But photosynthesis genes (e.g., *PSBO* (oxygen-evolving enhancer protein 1), *RBCS* (ribulose bisphosphate carboxylase small chain), and *FBA2* (fructose-bisphosphate aldolase 1)) in cotton were up-regulated to coordinate the photosynthetic process. Furthermore, cotton seedlings differentially regulated key biosynthesis and signaling components of phytohormonal pathways including abscisic acid, indoleacetic acid and gibberellin. This study elucidates the significant gene expression of drought-responsive transcriptional networks and relevant physiological response in cotton seedlings and offers a theoretical basis for developing water-saving irrigation strategies.

## 1. Introduction

Drought is one of the most severe climate disasters that affect human food security, hindering water absorption in plants, restricting plant growth, weakening photosynthesis, affecting agricultural production and threatening ecosystem stability [1,2]. Over the past decade, the total losses in crop production due to drought have reached approximately USD 30 billion [3]. With the growth of the population, the increasing demand for agricultural water, and the decreasing availability of freshwater, the impact of drought on agricultural production has been exacerbated, posing challenges to human society and the ecological environment [4].

The resistance mechanisms of plants under drought stress involve physiological, biochemical and genetic aspects. These mechanisms enable plants to withstand severe dehydration, regulate their growth periods to avoid water stress, and maintain important physiological processes [5,6]. Plants have developed a series of physiological and biochemical pathways and molecular mechanisms to cope with drought stress. For example, plants regulate stomatal opening and closing to control water transpiration [7,8]. In addition, they accumulate osmoregulatory substances such as proline and soluble sugars to maintain intracellular osmotic pressure and prevent water loss [9]. Moreover, plants enhance antioxidant activity, including superoxide dismutase activity and peroxidase activity, to mitigate damage [10]. The drought resistance of plants is influenced by genetic factors and gene expression [11,12]. Plants regulate gene expression to influence the functions of related proteins in response to drought stress.

Recent advancements in transcriptomics and Quantitative Real-Time Polymerase Chain Reaction (qRT-PCR) verification techniques have enabled the rapid identification of gene expression changes in plants under different physiological conditions [13,14]. These technologies play a crucial role in analyzing the molecular response mechanisms of plants under drought stress. Some transcriptome sequencing studies have identified genes related to drought resistance in plants. These genes can encode proteins for hormone signal transduction, the accumulation of osmolytes, membrane permeability, ion transport, and redox reactions [12,15,16]. These findings suggest that genetic and epigenetic changes in gene expression can ultimately enhance the drought resistance of plants.

Cotton (*Gossypium* spp.) is an annual herbaceous plant or perennial shrub of the genus *Gossypium* in the *Malvaceae* family [17]. As an important cash crop, cotton has shown that appropriate drought stress can enhance the adaptability of plants to drought from seed germination to seedling stage. It is vital to clarify the response mechanism of cotton seedlings under drought stress and manage them effectively to ensure the growth and yield of cotton. At present, most of the studies on the drought resistance mechanism of cotton only focus on exploring drought-resistant genes and verifying their functions [18,19,20]. However, there are few reports on the relationship between the physiological changes and the DEGs of cotton under drought stress [21,22,23].

Therefore, in this study, cotton seedlings were cultivated and subjected to mild, moderate, and severe drought stress treatments. Simultaneously, the transcriptome data of the seedlings under mild and severe stress treatments were analyzed to search DEGs. The objective of the study was to explore the impacts of drought stress on the osmotic regulation, antioxidant system, photosynthetic properties, and hormones in cotton seedlings. Furthermore, it also elucidated how these genes regulate the stress-resistant processes of cotton seedlings at the molecular level. This study contributes to revealing the adaptation mechanisms of cotton seedlings to drought stress from macroscopic to microscopic levels. And it provides a theoretical basis for formulating water-saving irrigation strategies in cotton fields.

## 2. Results

### 2.1. Evaluation and Validation of RNA-Seq

#### 2.1.1. Sequencing Data Analysis

As shown in Table 1 and Table 2, a total of 58.79 Gb of high-quality clean data were obtained, with an average of 5.36 Gb per sample and Q30 base percentages all above 90%. These results indicate that the sequencing is highly accurate and the data are suitable for subsequent in-depth analyses. Moreover, each sample yielded at least 35,766,806 clean reads, and the mapping rates to the reference genome ranged from 93.77% to 96.17%, demonstrating a high utilization efficiency of the transcriptomic data.

#### 2.1.2. Correlation Analysis Between Samples

**Figure 1:**
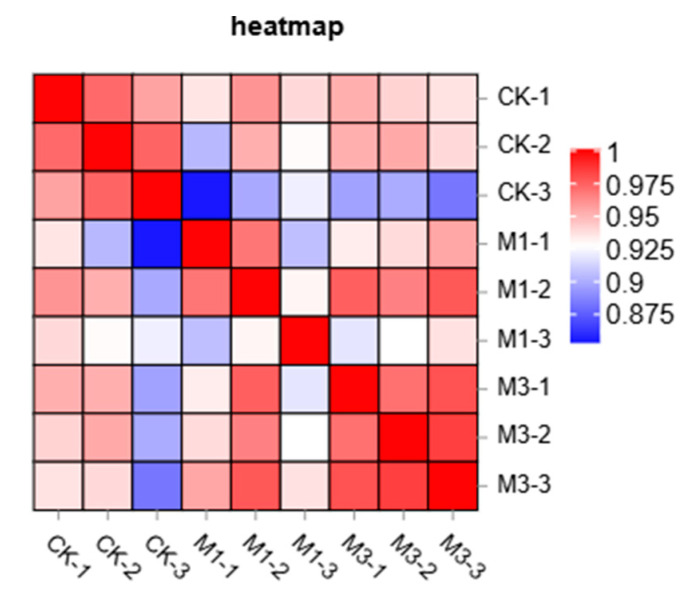
Heatmap of correlation analysis between samples. The CK group comprised CK-1, CK-2, and CK-3; the M1 group comprised M1-1, M1-2, and M1-3; and the M3 group comprised M3-1, M3-2, and M3-3.

Any two samples were taken to calculate Pearson’s correlation coefficient. As shown in Figure 1, the mean Pearson correlation coefficients within each treatment group exceeded 0.9, indicating a high level of similarity among replicates. In contrast, correlations between the control and M1 or M3 were moderate, reflecting a certain degree of divergence. These patterns of similarity and difference among samples provide a solid basis for further analysis.

### 2.2. KEGG (Kyoto Encyclopedia of Genes and Genomes) Metabolic Pathway Analysis of DEGs in Cotton Seedings Under Drought Stress

**Figure 2:**
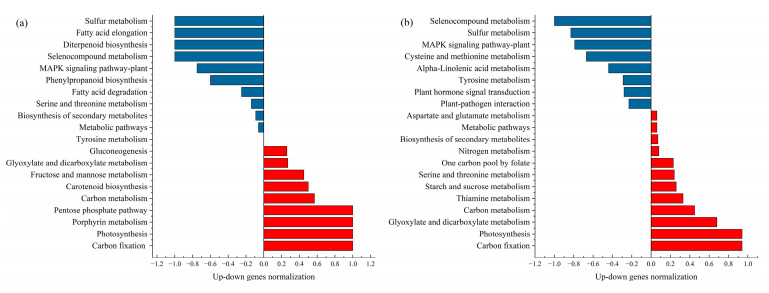
Compared with the control, the number and expression of DEGs in the KEGG metabolic pathways of cotton seedlings under mild (**a**) and severe (**b**) stress. In the figure, blue indicates down-regulation, while red indicates up-regulation.

KEGG functional annotation and pathway significant enrichment analyses were conducted on 4s171 DEGs. Under mild drought stress (Figure 2a), the up-regulated DEGs were predominantly enriched in pathways such as “Carbon fixation in photosynthetic organisms”, “Photosynthesis”, “Porphyrin metabolism”, and the “Pentose phosphate pathway”. The down-regulated DEGs were mainly concentrated in “Selenocompound metabolism”, “Diterpenoid biosynthesis”, “Fatty acid elongation”, and “Sulfur metabolism”.

In the case of severe drought stress (Figure 2b), the up-regulated DEGs were enriched in “Carbon fixation in photosynthetic organisms” and “Photosynthesis”. On the other hand, the down-regulated DEGs were mainly found to be enriched in the “MAPK signaling pathway-plant”, “Sulfur metabolism”, and “Selenocompound metabolism”.

Based on the above analysis, a detailed analysis was conducted on the DEGs related to the physiological indicators, photosynthesis, and hormone metabolic pathways of cotton seedlings.

### 2.3. An Analysis of the Effects of Drought Stress on the Physiological Indicators of Cotton Seedlings and the DEGs in Related Metabolic Pathways

#### 2.3.1. The Physiological Indicators of Cotton Seedlings Under Drought Stress

**Figure 3:**
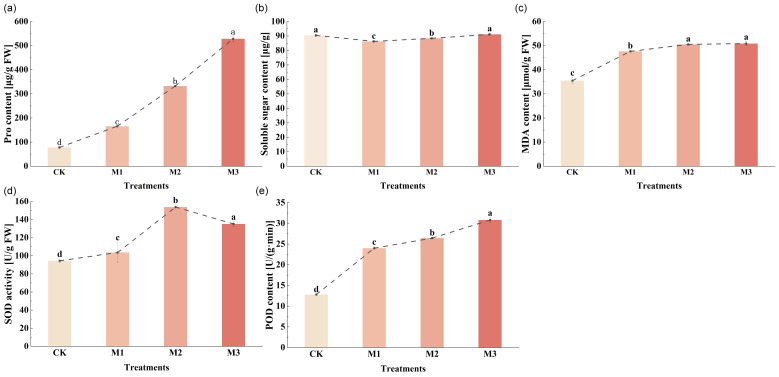
Changes in physiological indicators of cotton seedlings under drought stress, including proline content (**a**), soluble sugar content (**b**), malondialdehyde content (**c**), superoxide dismutase activity (**d**), and peroxidase content (**e**). Different letters indicate significant differences between groups, while the same letters indicate no significant difference. Three biological replicates were used in the statistical analysis.

The proline (Pro) content in cotton seedling leaves exhibited a progressive and significant increase with escalating stress levels, with the M3 treatment showing the highest Pro accumulation that was statistically superior to other treatments (Figure 3a). This pattern demonstrates severe water stress impact. Furthermore, the soluble sugar content displayed an initial decline followed by recovery under drought stress (Figure 3b), potentially attributable to the regulation of sugar metabolism-related genes.

As illustrated in Figure 3c, malondialdehyde (MDA) content showed a marked gradual elevation in cotton leaves, peaking in the M3 treatment, indicative of substantial membrane system damage. Correspondingly, while superoxide (SOD) activity initially increased and then decreased (Figure 3d,e), all drought-stressed groups maintained significantly higher SOD activity than the control, likely reflecting reactive oxygen species-induced activation of the antioxidant defense system. Peroxidase (POD) levels demonstrated a consistent and significant upward trend, reaching maximum values in the M3 treatment. This enhanced POD activity presumably functions to scavenge hydrogen peroxide and mitigate oxidative injury in cotton plants.

#### 2.3.2. Analysis of DEGs in Pathways Related to Physiological Indicators of Cotton Seedlings Under Drought Stress

**Figure 4:**
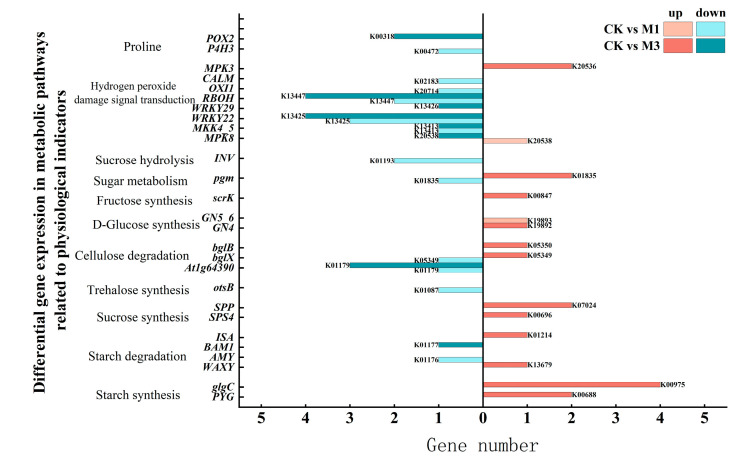
Compared with the control, DEGs of metabolic pathways related to physiological indicators of cotton seedlings under mild and severe drought stress.

As shown in Table 3 and Figure 4, in the arginine and proline metabolic pathways, DEGs related to proline degradation were down-regulated, aiding osmotic pressure balance under stress.

The starch and sucrose metabolic pathways relate to the production of soluble sugars in plants. Under mild stress, only DEGs related to D-glucose synthesis were up-regulated. In contrast, under severe stress, DEGs related to the synthesis of starch, sucrose, fructose, glucose, and sugar metabolism were all up-regulated. Evidently, under severe stress, cotton reprograms its sugar metabolism to prioritize sugar production and may actively induce sugar synthesis.

In the MAPK signaling pathway, MKK4/5 (mitogen-activated protein kinase kinase 4/5) activation is essential for stress defense. MKK4/5 regulates reactive oxygen species (ROS) production by phosphorylating WRKY22 (WRKY transcription factor 22), which influences OXI1 (serine/threonine-protein kinase OXI1) and RBOH (respiratory burst oxidase). Under mild stress, the down-regulation of DEGs, related to these proteins, contributed to reduce ROS production. Under severe stress, more down-regulated DEGs were enriched in these proteins. This indicated that cotton mitigates stress by suppressing ROS accumulation.

### 2.4. An Analysis of the Effects of Drought Stress on the Photosynthesis of Cotton Seedlings and the DEGs in Related Metabolic Pathways

#### 2.4.1. Changes in Chlorophyll Content and Analysis of DEGs in Cotton Seedlings Under Drought Stress

**Figure 5:**
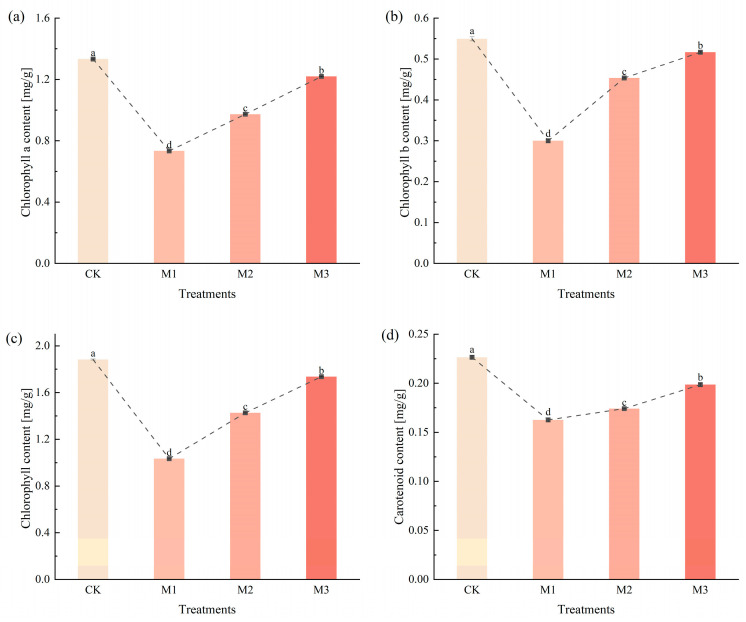
Changes in chlorophyll content of cotton seedlings under drought stress, including chlorophyll a content (**a**), chlorophyll b content (**b**), total chlorophyll content (**c**), and carotenoid content (**d**). Different letters indicate significant differences between groups, while the same letters indicate no significant difference. Three biological replicates were used in the statistical analysis.

As demonstrated in Figure 5, chlorophyll a, chlorophyll b, total chlorophyll content and carotenoid content were decreased. These changes indicated that drought stress inhibited chlorophyll synthesis and had a detrimental effect on the absorption and conversion of light energy in cotton leaves.

**Figure 6:**
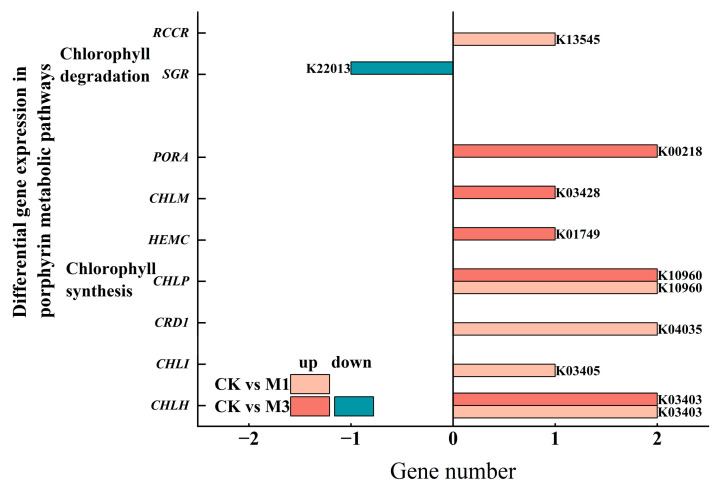
Compared with the control, DEGs in the porphyrin metabolic pathways in cotton seedlings under mild and severe drought stress.

The porphyrin pathway controls chlorophyll production. Under mild stress, eight DEGs for chlorophyll synthesis and degradation were up-regulated, with degradation surpassing synthesis (Table 4 and Figure 6). Under severe stress, the number of synthesis DEGs increased while degradation DEGs decreased (Figure 6), indicating plants might boost chlorophyll synthesis to maintain photosynthesis under stress.

#### 2.4.2. Changes in Gas Exchange Parameters and Analysis of DEGs in Photosynthesis Metabolic Pathway in Cotton Seedlings Under Drought Stress

**Figure 7:**
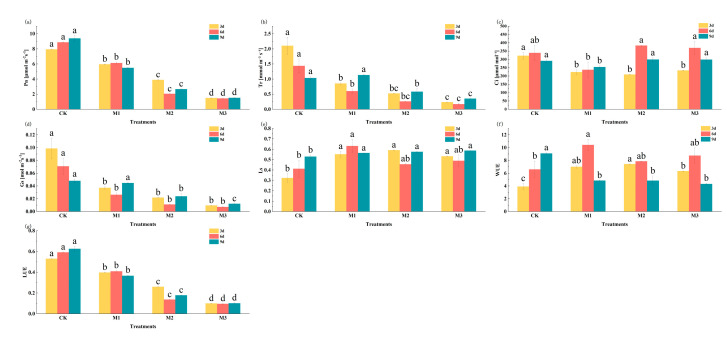
Changes in gas exchange parameters of cotton seedlings under drought stress, including the net photosynthetic rate (**a**), transpiration rate (**b**), intercellular carbon dioxide concentration (**c**), stomatal conductance (**d**), stomatal limiting values (**e**), water use efficiency (**f**), light use efficiency (**g**). Different letters indicate significant differences among treatment groups on the same day, whereas the same letter indicates no significant difference. Three biological replicates were used in the statistical analysis.

Gas exchange parameters are commonly used to indicate plant photosynthetic capacity. As shown in Figure 7, after 9 days of drought stress, the parameters of stomatal conductance (Gs), net photosynthetic rate (Pn), light use efficiency (LUE) and water use efficiency (WUE) progressively decreased with increasing stress intensity. Compared with the control, Gs, Pn, the transpiration rate (Tr), LUE and WUE showed significant reductions under both moderate and severe stress treatments, while confidence intervals (Cis) and stomatal limitation (Ls) increased, with Ls exhibiting a significant rise. These changes indicated that drought stress inhibited leaf photosynthesis and suppressed cotton growth.

**Figure 8:**
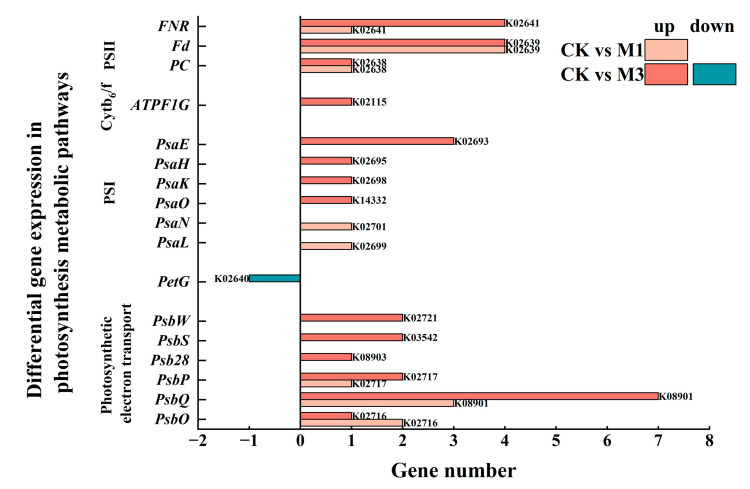
Compared with the control, DEGs in the photosynthesis metabolic pathway in cotton seedlings under mild and severe drought stress.

As shown in Figure 8 and Table 5, numerous DEGs in the photosynthesis metabolic pathway were up-regulated under stress. And the number of DEGs under severe stress was greater than that under mild stress. Among them, as shown in Table 6, the up-regulated expression of *PSBO* and *PSBP1* (oxygen-evolving enhancer protein 2) in the photosynthetic electron transporter was associated with the stabilization of the structure and function of the oxygen-evolving complex (OEC) and improvement in the activity and stability of Photosystem II (PSII). Similarly, the up-regulated expression of *PETE* (plastocyanin) and *PETH* (ferredoxin—NADP reductase) in PSII was likely involved in enhancing photosynthetic electron transport and improving light energy conversion efficiency.

#### 2.4.3. Analysis of DEGs in Carbon Fixation Metabolic Pathway in Cotton Seedlings Under Drought Stress

**Figure 9:**
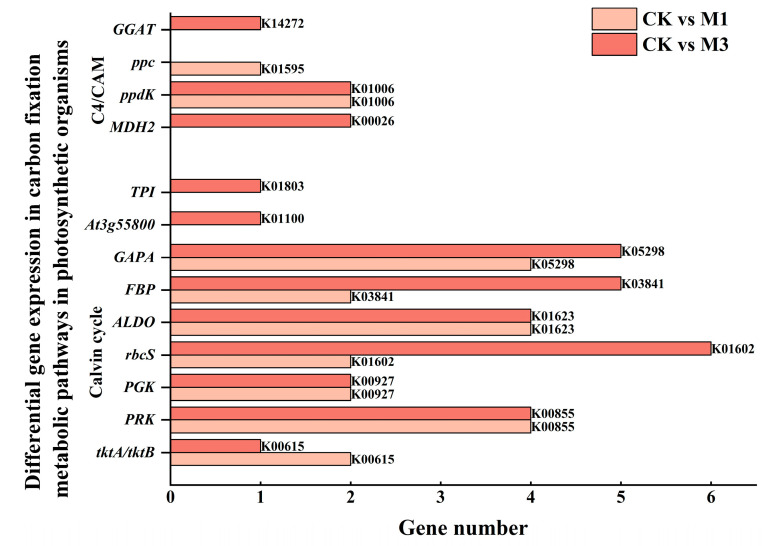
Compared with the control, DEGs in the carbon fixation metabolic pathway in cotton seedlings under mild and severe drought stress.

**Figure 10:**
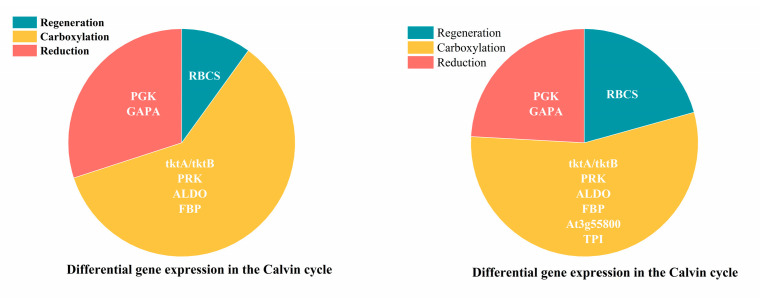
Compared with the control, DEGs in the carbon fixation metabolic pathway in cotton seedlings under mild (M1) and severe drought stress (M3).

As shown in Figure 9 and Table 7, DEGs in carbon fixation were up-regulated and particularly associated with the Calvin cycle. Among them, the expression of *RBCS*, *FBA2*, *GAPA*, and *TKL-2* were up-regulated (Table 6). This was associated with accelerated carbon dioxide fixation, the promotion of the synthesis of photosynthetic products, and an enhancement in the efficiency and stability of photosynthesis. And further analysis showed that DEGs were concentrated in the carboxylation stage (Figure 10).

### 2.5. An Analysis of the Effects of Drought Stress on Hormones in Cotton Seedlings and the DEGs in Related Metabolic Pathways

#### 2.5.1. Analysis of DEGs in Abscisic Acid-Related Metabolic Pathways in Cotton Seedlings Under Drought Stress

**Figure 11:**
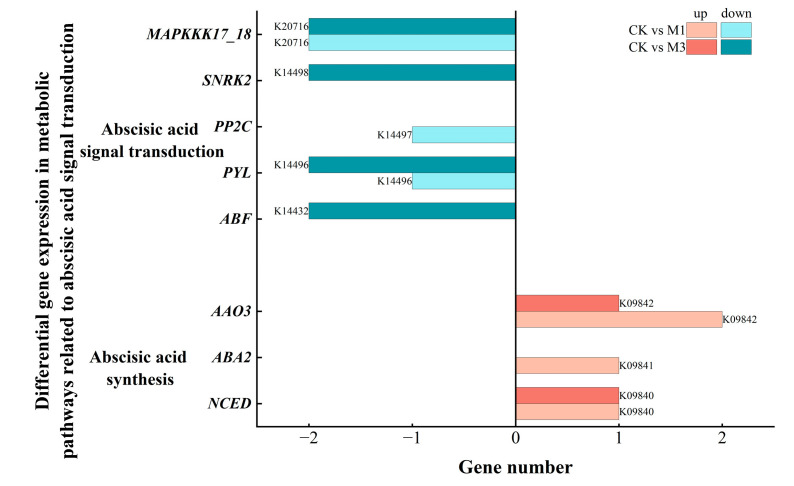
Compared with the control, DEGs in the ABA-related metabolic pathways in cotton seedlings under mild (M1) and severe drought stress (M3). Eight genes were differentially expressed.

Abscisic acid (ABA) is crucial for stress response. Under stress, DEGs in the ABA synthesis pathway were up-regulated and mainly enriched in *AAO3* (abscisic-aldehyde oxidase), *ABA2* (xanthoxin dehydrogenase), and *NCED* (9-cis-epoxycarotenoid dioxygenase) (Figure 11 and Table 8). This contributed to increased ABA levels. In contrast, DEGs in the ABA signal transduction were down-regulated and mainly were enriched in *MAPKKK17/18* (mitogen-activated protein kinase kinase kinase 17/18), *SNRK2* (serine/threonine-protein kinase SRK2), *PP2C* (protein phosphatase 2C), *PYL* (abscisic acid receptor PYR/PYL family), and *ABF* (ABA responsive element binding factor). This showed that the pathway was inhibited under stress.

#### 2.5.2. Analysis of DEGs in Indole-3-Acetic Acid-Related Metabolic Pathways in Cotton Seedlings Under Drought Stress

**Figure 12:**
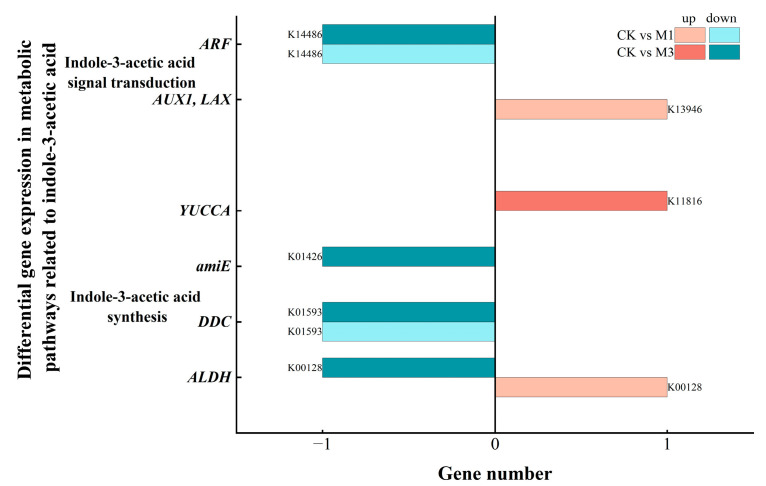
Compared with the control, DEGs in the IAA-related metabolic pathways in cotton seedlings under mild (M1) and severe drought stress (M3). Seven genes were differentially expressed.

The synthesis of IAA in plants is regulated by multiple enzymes. As shown in Figure 12 and Table 8, under stress, numerous DEGs in the IAA synthesis pathway were down-regulated and enriched in *amiE*, *DDC*, and *ALDH*. Similarly, in the signal transduction pathway, *AUX1/LAX* encodes a transport carrier protein for IAA. *ABF* is involved in the negative regulation of signal transduction. The up-regulation of DEGs enriched in *AUX1/LAX* and the down-regulation of DEGs enriched in *ABF* occurred under stress. This contributed to the regulation of IAA in the physiological responses of cotton seedlings.

#### 2.5.3. Analysis of DEGs in Gibberellin-Related Metabolic Pathways in Cotton Seedlings Under Drought Stress

**Figure 13:**
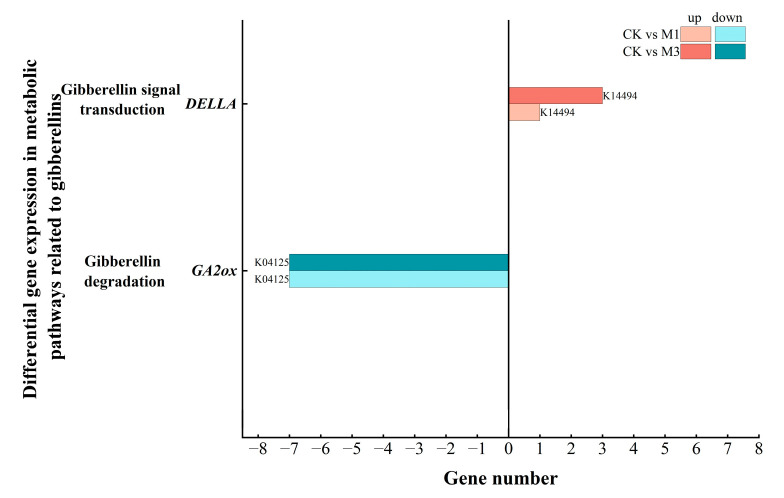
Compared with the control, DEGs in the GA-related metabolic pathways in cotton seedlings under mild (M1) and severe drought stress (M3). Two genes were differentially expressed.

Gibberellin (GA) promotes plant growth processes like seed germination and internode elongation. GA2ox is a key enzyme for gibberellin degradation and DELLA protein is a negative regulator in the signal transduction pathway. As shown in Figure 13 and Table 8, the DEGs were down-regulated and mainly enriched in *GA2ox*. And among them, the expression of *GA2OX1* was down-regulated (Table 6 and Figure 13). But the up-regulated DEGs were mainly enriched in DELLA proteins.

### 2.6. Verification of DEGs by RT-qPCR

**Figure 14:**
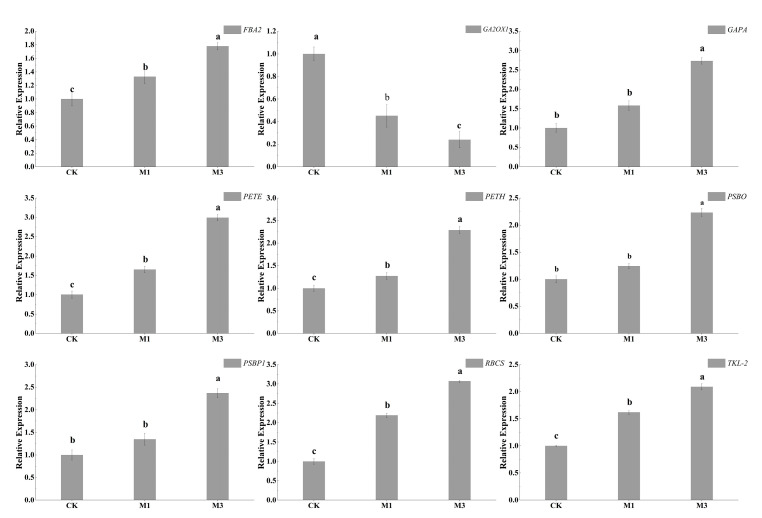
Results of gene expression validation by qRT-PCR. Relative gene expression was calculated using the 2−ΔΔCt method, with UBC28 as the endogenous control. Three technical replicates were performed for each sample, exhibiting a significant correlation with RNA-Seq results (R = 0.51, *p* < 0.05, Pearson’s test). Different letters indicate significant differences among different treatment groups, while the same letter indicates no significant difference.

The expression levels of the screened differential genes under mild and severe drought stress were verified by qRT-PCR (Table 6). As shown in Figure 14, the expression levels of *PSBO*, *PSBP1*, *PETE*, *PETH*, *RBCS*, *FBA2*, *GAPA*, and *TKL-2* were up-regulated, while *GA2OX1* was down-regulated. These results corroborated the transcriptome findings, confirming data reliability.

## 3. Discussion

Plant cells experience a reduction in turgor due to water loss through cellular dehydration under drought stress [24]. The accumulation of osmoregulatory substances increases intracellular solute concentration, reduces osmotic potential, and enhances the water-holding capacity of cells under drought stress [25,26]. Compared with the control, down-regulated DEGs associated with proline catabolic enzymes (e.g., *POX2* and *P4H3*) correlated with elevated proline accumulation, suggesting suppressed degradation pathways [27,28]. This contributed to the increase in proline content [29]. In the sugar metabolism pathway (Figure 4), cotton seedlings were not severely affected under mild stress. They mainly enhanced the synthesis of glucose to meet the needs of basic physiological functions and reduced energy consumption in decomposing macromolecular polysaccharides. However, the investigation centered on the stress-induced biosynthesis and accumulation of soluble carbohydrates as a key osmoregulatory mechanism under severe stress [30]. Cotton seedlings exhibited the enhanced biosynthesis of hexoses (glucose and fructose), serving as both osmolytes and metabolic substrates to maintain various physiological activities. In general, the down-regulation of DEGs enriched in proline degradation proteins under stress contributed to significant proline accumulation (Figure 3a). In sugar metabolism, mild stress focused on glucose synthesis to meet basic physiological needs, while severe stress triggered the systemic accumulation of osmoprotective sugars through carbon flux redirection, balancing osmotic adjustment with energy preservation (Figure 3b). Notably, sugar accumulation and degradation do not represent completely independent processes but may occur simultaneously. On the one hand, sugar accumulation contributes to maintaining cellular osmotic potential, thereby playing an osmoregulatory role under stress conditions. This mechanism helps plant cells maintain water balance and enhances stress resistance [31,32]. On the other hand, sugar degradation provides energy for plant growth and development, while the intermediate metabolites generated can serve as precursors for the synthesis of other essential biomolecules to meet the demands of various plant tissues [33].

In a drought environment, plants produce a large amount of reactive oxygen species, triggering lipid peroxidation cascades in plasma membranes while simultaneously activating a coordinated antioxidant response involving enzymatic (SOD and POD) and non-enzymatic (ascorbate and glutathione) defense systems to restore cellular redox homeostasis [34,35]. The MDA content significantly increased (Figure 3c), indicative of membrane lipid peroxidation, which subsequently damaged the plant cell membrane system under stress conditions [36,37]. Therefore, in the MAPK signaling pathway (Figure 4), some DEGs related to reactive oxygen species were down-regulated under mild stress. Consequently, this inhibited the production of reactive oxygen species and regulated the activity of antioxidant enzymes [38,39,40]. Under severe stress, the number of down-regulated DEGs related to reactive oxygen species was increased, which coordinated with the increase in SOD and POD activity [41] (Figure 3d,e). This enhanced ROS scavenging capacity through the synergistic suppression of oxidative burst and amplification of detoxification pathways [42]. Therefore, this contributed to maintaining cell stability and reducing the degree of oxidative damage in cotton [36].

The chlorophyll content in leaves is an important indicator to reflect the photosynthetic capacity of plant leaves [43]. The chlorophyll and carotenoid contents in the leaves of cotton seedlings decreased (Figure 5). Drought stress affected the absorption and conversion of light energy in cotton [44]. Gas exchange parameters are commonly used to assess photosynthetic efficiency. Drought stress significantly impaired photosynthetic performance in cotton seedlings, and consequently, WUE, Pn, Tr and Gs decreased (Figure 7), collectively resulting in growth inhibition through carbon assimilation suppression [44].

Although photosynthetic capacity was inhibited, cotton seedlings adjusted their photosynthetic metabolism pathways to provide the necessary energy and substances for growth (Figure 8 and Table 5). In the porphyrin metabolic pathway (Figure 6), severe stress induced the increase in chlorophyll synthesis-related DEGs. Meanwhile the down-regulation of chlorophyll catabolism-associated DEGs was enriched in the senescence pathway to sustain photosynthetic competence under severe stress [45]. Moreover, in the photosynthesis metabolic pathway, the up-regulation of *PSBO* and *PSBP1* in photosynthetic electron transporter stabilized the structure and function of OEC and improved the activity and stability of PSII [46]. Similarly, *PETE* and *PETH* in PSII improved photosynthetic electron transport and light energy conversion efficiency [47]. Furthermore, in the carbon fixation metabolic pathway, the up-regulation of *RBCS*, *FBA2*, *GAPA*, and *TKL-2* accelerated CO_2_ fixation, promoted the synthesis of photosynthetic products, and enhanced the efficiency and stability of photosynthesis [48]. Evidently, although drought led to stomatal closure, cotton increased the utilization rate of CO_2_ to enhance its survival ability under stress condition.

In addition, under different stress treatments, cotton seedlings precisely regulated hormone-related genes (Table 8) to modulate the levels and functions of hormones (ABA, IAA and GA) under stress conditions. The up-regulation of DEGs, enriched in the ABA synthesis pathway, helped to increase ABA levels [49,50,51], but the signal transduction pathways were inhibited. This might have been related to the negative feedback regulation when the ABA content was too high. This enabled cotton to sustain stress-responsive ABA biosynthesis while mitigating ABA hyperaccumulation-induced growth arrest [52,53]. Meanwhile, under different stress treatments, cotton selectively regulated the synthesis pathway of IAA [54,55]. In the IAA signal transduction pathway, the up-regulation of DEGs, enriched in *AUX1/LAX*, facilitated the absorption and transportation of IAA [56]. And the down-regulation of DEGs, enriched in *ABF*, could more effectively activate IAA signaling pathway to enhance related physiological responses [57]. In addition, in GA-related pathways, the down-regulation of *GA2OX1* helped maintain a relatively high level of GA content in cells [58]. But the up-regulated DEGs in gibberellin signal transduction were mainly enriched in a negative regulatory protein called DELLA (Figure 13). This might be related to the regulatory pattern mediated by ABA-GA hormonal crosstalk [59], promoting the enrichment of up-regulated DEGs in DELLA-dependent stress-responsive genes. ABA and GA exhibit pronounced antagonism in plant growth, development, and stress responses. ABA typically accumulates under adverse conditions to inhibit plant growth—such as seed germination and seedling elongation—whereas GA promotes these very processes [60]. ABA inhibits the GA signaling pathway by suppressing the GA-dependent degradation of DELLA proteins [61,62].

**Figure 15:**
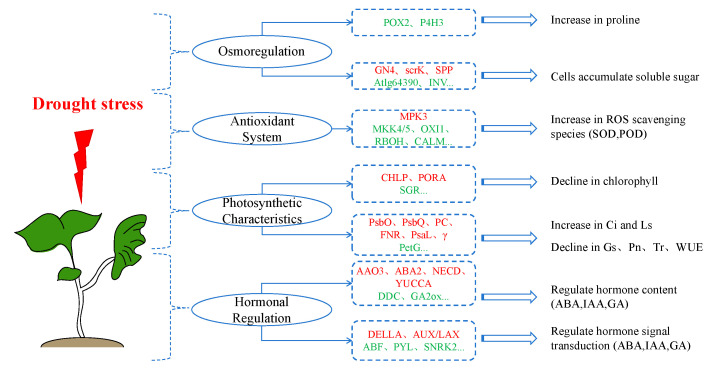
Flowchart of analysis of physiological responses and transcriptome of cotton seedlings to drought stress.

As shown in Figure 15, this study mainly explored the effects of stress on the osmotic adjustment, antioxidant system, photosynthetic characteristics, and hormonal regulation of cotton seedlings. Meanwhile, it systematically characterized stress-responsive transcriptional networks and how they synergistically interact to regulate the growth and stress resistance processes of cotton seedlings.

## 4. Materials and Methods

### 4.1. Plant Cultivation

The experiment used “ZhongMian 113” cotton seeds sourced from HuaXing Farm in the Changji City of Xinjiang (Xinjiang Zhongnongyou Cotton Industry Co., Ltd., Shihezi, China). Uniform seeds were sterilized, soaked at 24 °C (65–70% humidity, dark) until roots reached about 1 cm, and then sown and cultured in a light incubator (24 °C, 14 h/10 h light/dark cycle). After developing two cotyledons, seedlings were transplanted for stress treatment.

### 4.2. Experimental Design

**Figure 16:**
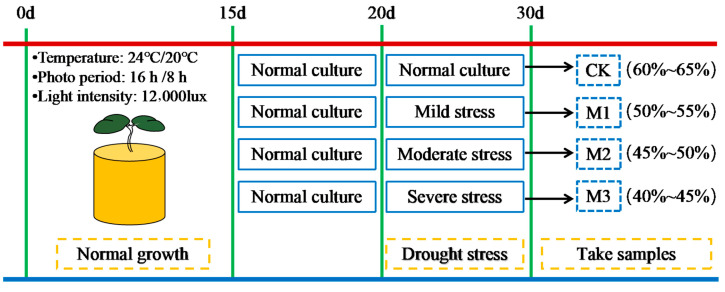
Experimental design.

As in Figure 16, three drought stress treatments were set: normal culture (60–65% field moisture), mild stress (50–55% field moisture), moderate stress (45–50% field moisture), and severe stress (40–45% field moisture). Soil moisture was regularly monitored with a soil moisture meter, and each treatment had three replicates.

### 4.3. Determination of Physiological Indicators

After treatment, sampling was conducted three hours after the onset of illumination (simulating 9–10 a.m.). The leaves samples were taken and frozen in liquid nitrogen and then were stored in −80 °C. The methods for measuring the physiological indicators of the samples were referenced from Gao [63] and all measurements were performed in triplicate.

#### 4.3.1. Determination of Malondialdehyde Content

Weigh 2 g of fresh leaves and add a small amount of quartz sand along with 5 mL of 10% trichloroacetic acid. Grind the mixture to form a homogenate, and then add an additional 15 mL of 10% trichloroacetic acid for further grinding. Centrifuge the homogenate at 4000 revolutions per minute (r/min) for 10 min. The supernatant obtained is the MDA extract. Next, transfer 2 mL of the extract into a test tube and add 2 mL of 0.6% thiobarbituric acid solution. Mix the contents well, and then heat the mixture in a boiling water bath for 15 min. After heating, cool the mixture rapidly, and centrifuge it again at 4000 r/min for 3 min to separate the supernatant. Finally, measure the absorbance values of the supernatant at wavelengths of 532 nm, 600 nm, and 450 nm, using distilled water as the blank control. Calculate the MDA content based on these absorbance measurements.

#### 4.3.2. Determination of Proline Content

Fresh leaves were cut into pieces, and 0.5 g of them was weighed and placed in a large test tube. Then, 5 mL of 3% sulfosalicylic acid was added, and the tube mouth was covered with a glass stopper. The mixture was extracted in a boiling water bath for 15 min, with frequent shaking during the extraction process. After cooling, the mixture was filtered into a clean test tube, and the filtrate served as the proline extract. In total, 2 mL of the extract was pipetted into a clean stoppered test tube, followed by the addition of 2 mL of glacial acetic acid and 2 mL of acidic ninhydrin reagent. The mixture was heated in a boiling water bath for 30 min, resulting in a red-colored solution. After cooling, 5 mL of toluene was added, and the mixture was vigorously shaken for 30 s. After standing to allow phase separation, the upper layer of proline–toluene solution was aspirated, and its absorbance was measured at 520 nm. The proline content in the sample was calculated based on the proline standard curve.

#### 4.3.3. Determination of Superoxide Dismutase Activity

SOD activity was determined using the nitroblue tetrazolium (NBT) photoreduction method. Briefly, 0.5 g of leaf tissue was homogenized in 5 mL of 50 mM phosphate buffer (pH 7.8) in an ice bath, followed by centrifugation at 10,000 r/min for 10 min at 4 °C. The resulting supernatant was collected as the enzyme extract. The reaction mixture (total volume 3.0 mL) contained 1.6 mL of 50 mM phosphate buffer (pH 7.8), 0.3 mL of 20 μM riboflavin, 0.3 mL of 130 mM methionine, 0.3 mL of 100 μM EDTA, 0.3 mL of 750 μM NBT, and 0.12 mL of enzyme extract (the control reaction used 0.12 mL phosphate buffer instead of enzyme extract). The reaction was initiated by exposing the mixture to 4000 Lux light for 25 min at 25 °C, after which the absorbance was measured at 560 nm. One unit of SOD activity (U) was defined as the amount of enzyme required to inhibit 50% of NBT photoreduction under the assay conditions. Enzyme activity was expressed as units per gram fresh weight (U/g FW). All measurements were performed in triplicate.

#### 4.3.4. Determination of Peroxidase Content

The POD content was determined using the guaiacol method. Briefly, 0.5 g of leaf tissue was homogenized with a small amount of quartz sand and calcium carbonate powder in distilled water in an ice bath. The homogenate was diluted to 50 mL with distilled water and centrifuged at 12,000 r/min for 30 min at 4 °C. The supernatant was collected as the enzyme extract. The reaction system (total volume 5.0 mL) consisted of 0.5 mL of 0.1% guaiacol, 3.45 mL of distilled water, 0.5 mL of 0.18% H_2_O_2_ (the control reaction used 0.5 mL distilled water instead of H_2_O_2_), and 0.5 mL of enzyme extract. After thorough mixing, the reaction was allowed to proceed for 10 min at 25 °C and then terminated by adding 0.1 mL of 5% metaphosphoric acid. The absorbance change rate was immediately measured at 470 nm using a UV-Vis spectrophotometer (752G; Shanghai Instrument Electric Analytical Instruments Co., Ltd.; Shanghai, China). One unit of POD activity was defined as the amount of enzyme causing an absorbance change of 0.01 per minute under the assay conditions. Enzyme activity was expressed as units per gram fresh weight (U/g FW). All measurements were performed in triplicate.

### 4.4. Determination of Chlorophyll Content and Hormone

Chlorophyll content was measured according to the method of Zhang et al. [64], including chlorophyll a, chlorophyll b and carotenoid. The chlorophyll content in leaves was determined using the ethanol extraction method. Fresh leaf tissue (0.2 g, excluding midribs) was cleaned, cut into small pieces, and ground in a mortar with a small amount of quartz sand, calcium carbonate powder, and 2–3 mL of 95% ethanol under ice bath conditions until the tissue turned white and a homogeneous slurry was obtained. The homogenate was filtered through ethanol-moistened filter paper into a 25 mL brown volumetric flask. The mortar and residue were repeatedly rinsed with ethanol until no green color remained on the filter paper, and the volume was finally adjusted to 25 mL with ethanol and mixed thoroughly. The extract was transferred to a cuvette, and absorbance was measured at 665 nm, 645 nm, and 470 nm using a UV-Vis spectrophotometer (752G; Shanghai Instrument Electric Analytical Instruments Co., Ltd.; Shanghai, China), with 95% ethanol serving as the blank control. The samples were sent to Shandong Guocangjian Biotechnology Co., Ltd., Taian, China, for testing. The concentrations of IAA, ABA, and GA in the extracted and concentrated leaf samples were measured using liquid chromatography–mass spectrometry (LC-MS) technology.

### 4.5. Measurement of Gas Exchange Parameters

The photosynthesis properties of cotton leaves were measured by using an Li-6400 photosynthesis measurement system (Li-6400; LI-COR Inc.; Lincoln, NE, USA), including Pn, Gs, LUE, WUE, Ls, Ci and Tr. Each treatment included three biological replicates.

### 4.6. RNA Sequencing and Data Analysis

Sampling was conducted three hours after the onset of illumination (simulating 9–10 a.m.). The cotton leaves of normal culture, mild stress and severe stress were sent to Guangzhou Kidio Biotechnology Co., Ltd., Guangzhou, China, for RNA-seq. The experimental procedures involved removing rRNA from the samples using a standard kit to enrich mRNA, assessing RNA integrity using the Agilent 2100 Bioanalyzer (Agilent 2100 Bioanalyzer; Agilent Technologies, Inc.; Santa Clara, CA, USA), and selecting cDNA fragments by size, specifically using AMPure XP beads (AMPure XP beads; Beckman Coulter, Inc.; Brea, CA, USA) to select cDNA fragments around 200 bp, followed by PCR amplification and purifying the PCR products again with AMPure XP beads to obtain the final PCR library. Each treatment has three biological replicates and there are nine samples in total. Then, the obtained data were analyzed.

### 4.7. Real-Time Quantitative PCR

Sampling was conducted three hours after the onset of illumination (simulating 9–10 a.m.). The cotton leaves of normal culture, mild stress and severe stress were sent to Guangzhou Kidio Biotechnology Co., Ltd., China, for RT-qPCR. The detailed procedure was as follows: After verifying the accuracy of the samples, nucleic acid extraction was performed. Following the normalization of concentration, 12 µL of RNA (total amount 500 ng–2 µg) was added for reverse transcription. Subsequently, genomic DNA was removed (12 µL RNA was mixed with 4 µL 4× gDNA Wiper Mix, briefly centrifuged, and reacted at 42 °C for 2 min) to obtain the reaction mixture. For this reaction mixture, cDNA was synthesized by reverse transcription (16 µL of the genomic DNA-free reaction mixture was mixed with 4 µL 5× qRT Super Mix II, briefly centrifuged, and reacted at 50°C for 15 min, followed by termination at 85 °C for 2 min). Then, based on the gene sequence information, qPCR amplification primers were designed using the primer design software Primer 5 and synthesized by the primer synthesis department (Table 9). Subsequently, quantitative real-time PCR (qPCR) was conducted using the reaction system specified in Table 10.

### 4.8. Data Analysis

SPSS 25.0 software [65] was used for statistical analysis. The experimental data were presented as mean ± standard error. The significance of differences in a specific indicator between treatments was assessed by employing a single-factor ANOVA. The specific steps are as follows: First, input the data into SPSS and conduct a normality test. Then click on “Analyze” > “Compare Means” > “One-Way ANOVA.” Next, click on “Options,” check “Descriptive” and “Homogeneity of Variance Test,” and finally, click “Continue” to return to the main dialog box. The LSD test was used for multiple comparisons. Origin 2021 software [66] was used to generate plots of the data.

## 5. Conclusions

Under drought stress, cotton seedlings maintained homeostasis and growth by integrating physiological and transcriptional responses. For osmotic adjustment, the transcriptional repression of proline catabolism-associated DEGs reduced enzymatic degradation capacity, resulting in the accumulation of proline. And cotton seedlings demonstrated regulation patterns that varied with drought intensity. In terms of antioxidation, oxidative stress triggered by drought stress induced MDA accumulation, causing damage to the membrane system. Consequently, the redox imbalance activated coordinated antioxidant response systems, such as an increase in SOD and POD activity. Meanwhile, drought stress impaired the photosynthetic performance of cotton seedlings. But cotton seedlings exhibited compensatory metabolic reprogramming through regulating the expression of genes in chlorophyll synthesis, photosynthetic electron transport, and carbon fixation. Moreover, for hormone regulation, cotton could precisely regulate the genes related to hormones (ABA, IAA, and GA) and their signal transduction pathways to cope with drought stress. Future research should explore the interactions among various regulatory pathways through integrated systems biology approaches to unravel the mechanism of drought resistance in cotton. This will accelerate the development of cotton drought resistance through the predictive modeling of gene-pyramiding strategies. And this is beneficial for providing information in precision agriculture practices for sustainable yield stabilization under drought stress.

## Figures and Tables

**Table 1:** Statistical analysis of sample transcriptome sequencing data.

Samples	Clean Reads	CLean_Bases	GC Content	% ≥ Q30
CK (normal culture)-1	43,657,062	6,514,911,687	43.49%	91.81%
CK-2	41,758,086	6,232,287,901	43.71%	91.28%
CK-3	38,925,918	5,813,473,089	43.75%	92.10%
M1-1	37,720,786	5,616,674,520	43.30%	90.53%
M1-2	54,369,244	8,111,458,084	43.81%	92.04%
M1-3	43,414,136	6,481,368,190	43.90%	92.07%
M3-1	44,289,592	6,605,707,682	43.61%	91.77%
M3-2	35,961,940	5,367,231,914	43.72%	91.34%
M3-3	53,940,862	8,050,791,622	43.82%	92.80%

**Table 2:** Statistics of sequence alignment results between the sample sequencing data and the reference genome.

Samples	Clean Read Number	Total Mapped	Unique Mapped	Multiple Mapped
CK-1	43,507,538	41,842,721 (96.17%)	40,106,591 (92.18%)	1,736,130 (3.99%)
CK-2	41,512,474	39,801,003 (95.88%)	38,059,123 (91.68%)	1,741,880 (4.20%)
CK-3	38,740,216	37,245,056 (96.14%)	35,681,502 (92.10%)	1,563,554 (4.04%)
M1-1	37,515,964	35,178,416 (93.77%)	33,588,795 (89.53%)	1,589,621 (4.24%)
M1-2	54,016,576	51,767,192 (95.84%)	49,485,423 (91.61%)	2,281,769 (4.22%)
M1-3	43,182,614	41,068,568 (95.10%)	39,307,967 (91.03%)	1,760,601 (4.08%)
M3-1	44,085,140	41,683,531 (94.55%)	39,873,331 (90.45%)	1,810,200 (4.11%)
M3-2	35,766,806	33,894,337 (94.76%)	32,427,872 (90.66%)	1,466,465 (4.10%)
M3-3	53,671,742	51,261,221 (95.51%)	49,041,525 (91.37%)	2,219,696 (4.14%)

Note: Total Mapped: reads mapped to the reference genome; Unique Mapped: reads mapped to a unique location in the reference genome; Multiple Mapped: reads mapped to multiple locations in the reference genome.

**Table 3:** Compared with the control, the names of DEGs of metabolic pathways related to physiological indicators of cotton seedlings under mild and severe drought stress.

Pathway	K_ID	Gene	Gene Type
Sucrose and starch metabolism (ko00500)	K00688	*PYG*	*PYG, glgP;* glycogen phosphorylase [EC: 2.4.1.1]
	K00975	*glgC*	*glgC*; glucose-1-phosphate adenylyltransferase [EC: 2.7.7.27]
	K13679	*WAXY*	*WAXY*; granule-bound starch synthase [EC: 2.4.1.242]
	K01176	*AMY*	*AMY, amyA, malS*; alpha-amylase [EC: 3.2.1.1]
	K01177	*BAM1*	*E3.2.1.2*; beta-amylase [EC: 3.2.1.2]
	K01214	*ISA*	*ISA, treX*; isoamylase [EC: 3.2.1.68]
	K00696	*SPS4*	*E2.4.1.14*; sucrose-phosphate synthase [EC: 2.4.1.14]
	K07024	*SPP*	*SPP*; sucrose-6-phosphatase [EC: 3.1.3.24]
	K01193	*INV*	*INV, sacA*; beta-fructofuranosidase [EC: 3.2.1.26]
	K01087	*otsB*	*otsB*; trehalose 6-phosphate phosphatase [EC: 3.1.3.12]
	K01179	*At1g64390*	*E3.2.1.4*; endoglucanase [EC: 3.2.1.4]
	K05349	*bglX*	*bglX*; beta-glucosidase [EC: 3.2.1.21]
	K05350	*bglB*	*bglB*; beta-glucosidase [EC: 3.2.1.21]
	K00847	*scrK*	*E2.7.1.4*, scrK; fructokinase [EC: 2.7.1.4]
	K01835	*pgm*	*pgm*; phosphoglucomutase [EC: 5.4.2.2]
	K19892	*GN4*	*GN4*; glucan endo-1,3-beta-glucosidase 4 [EC: 3.2.1.39]
	K19893	*GN5/6*	*GN5/6*; glucan endo-1,3-beta-glucosidase 5/6 [EC: 3.2.1.39]
MAPK signaling pathway—plant (ko04016)	K20538	*MPK8*	*MPK8*; mitogen-activated protein kinase 8 [EC: 2.7.11.24]
	K13413	*MKK4/5*	*MKK4/5*; mitogen-activated protein kinase kinase 4/5 [EC: 2.7.12.2]
	K13425	*WRKY22*	*WRKY22*; WRKY transcription factor 22
	K13426	*WRKY29*	*WRKY29*; WRKY transcription factor 29
	K13447	*RBOH*	*RBOH*; respiratory burst oxidase [EC: 1.6.3.- 1.11.1.-]
	K20714	*OXI1*	*OXI1*; serine/threonine-protein kinase OXI1 [EC: 2.7.11.1]
	K02183	*CALM*	*CALM*; calmodulin
	K20536	*MPK3*	*MPK3*; mitogen-activated protein kinase 3 [EC: 2.7.11.24]
Arginine and proline metabolism (ko00330)	K00472	*P4H3*	*P4HA*; prolyl 4-hydroxylase [EC: 1.14.11.2]
	K00318	*POX2*	*PRODH, fadM, putB*; proline dehydrogenase [EC: 1.5.5.2]

**Table 4:** Compared with the control, the names of DEGs in the porphyrin metabolic pathways in cotton seedlings under mild and severe drought stress.

Pathway	K_ID	Gene	Gene Type
Porphyrin metabolism (ko00860)	K03403	*CHLH*	*chlH, bchH*; magnesium chelatase subunit H [EC: 6.6.1.1]
	K03405	*CHLI*	*chlI, bchI*; magnesium chelatase subunit I [EC: 6.6.1.1]
	K04035	*CRD1*	*E1.14.13.81, acsF, chlE*; magnesium-protoporphyrin IX monomethyl ester (oxidative) cyclase [EC: 1.14.13.81]
	K10960	*CHLP*	*chlP, bchP*; geranylgeranyl diphosphate/geranylgeranyl-bacteriochlorophyllide a reductase [EC: 1.3.1.83 1.3.1.111]
	K01749	*HEMC*	*hemC, HMBS*; hydroxymethylbilane synthase [EC: 2.5.1.61]
	K03428	*CHLM*	*bchM, chlM*; magnesium-protoporphyrin O-methyltransferase [EC: 2.1.1.11]
	K00218	*PORA*	*por*; protochlorophyllide reductase [EC: 1.3.1.33]
	K22013	*SGR*	*SGR, SGRL*; magnesium dechelatase [EC: 4.99.1.10]
	K13545	*RCCR*	*RCCR, ACD2*; red chlorophyll catabolite reductase [EC: 1.3.7.12]

**Table 5:** Compared with the control, the names of DEGs in the photosynthesis metabolic pathway in cotton seedlings under mild and severe drought stress.

Category	Protein Subunit	K_ID	Gene Type
PSII	PsbO	K02716	*psbO*; photosystem II oxygen-evolving enhancer protein 1
	PsbQ	K08901	*psbQ*; photosystem II oxygen-evolving enhancer protein 3
	PsbP	K02717	*psbP*; photosystem II oxygen-evolving enhancer protein 2
	Psb28	K08903	*psb28*; photosystem II 13kDa protein
	PsbS	K03542	*psbS*; photosystem II 22kDa protein
	PsbW	K02721	*psbW*; photosystem II PsbW protein
Cytb_6_/f	PetG	K02640	*petG*; cytochrome b6-f complex subunit 5
ATPase	γ	K02115	*ATPF1G, atpG*; F-type H+-transporting ATPase subunit gamma
PSⅠ	PsaL	K02699	*psaL*; photosystem I subunit XI
	PsaN	K02701	*psaN*; photosystem I subunit PsaN
	PsaO	K14332	*psaO*; photosystem I subunit PsaO
	PsaK	K02698	*psaK*; photosystem I subunit X
	PsaH	K02695	*psaH*; photosystem I subunit VI
	PsaE	K02693	*psaE*; photosystem I subunit IV
Photosynthetic electron transport	PC	K02638	*petE*; plastocyanin
	Fd	K02639	*petF*; ferredoxin
	FNR	K02641	*petH*; ferredoxin--NADP+ reductase

**Table 6:** Compared with the control, names of screened DEGs related to the physiological indicators, photosynthesis, and hormone metabolic pathways of cotton seedlings under mild and severe drought stress.

Gene ID	Symbol	K_ID	Description
GH_D13G1739	*GA2OX1*	K04125	PREDICTED: GA2ox2
GH_A11G1964	*PSBO*	K02716	PREDICTED: oxygen-evolving enhancer protein 1, chloroplastic-like
GH_A05G4010	*PSBP1*	K02717	PREDICTED: oxygen-evolving enhancer protein 2, chloroplastic-like
GH_D05G1861	*PETE*	K02638	PREDICTED: plastocyanin
GH_D11G1863	*PETH*	K02641	PREDICTED: ferredoxin—NADP reductase, leaf isozyme, chloroplastic
GH_A07G1943	*RBCS*	K01602	PREDICTED: ribulose bisphosphate carboxylase small chain, chloroplastic
GH_A13G0295	*FBA2*	K01623	PREDICTED: fructose-bisphosphate aldolase 1, chloroplastic-like
GH_A10G0699	*GAPA*	K05298	PREDICTED: glyceraldehyde-3-phosphate dehydrogenase A, chloroplastic-like
GH_D07G1598	*TKL-2*	K00615	PREDICTED: transketolase, chloroplastic

**Table 7:** Compared with the control, the names of DEGs in the carbon fixation metabolic pathway in cotton seedlings under mild and severe drought stress.

Pathway	K_ID	Gene	Gene Name
Carbon fixation in photosynthetic organisms (ko00710)	K00615	*tktA/tktB*	*E2.2.1.1, tktA, tktB*; transketolase [EC: 2.2.1.1]
	K00855	*PRK*	*PRK, prkB*; phosphoribulokinase [EC: 2.7.1.19]
	K00927	*PGK*	*PGK, pgk*; phosphoglycerate kinase [EC: 2.7.2.3]
	K01602	*rbcS*	*rbcS*; ribulose-bisphosphate carboxylase small chain [EC: 4.1.1.39]
	K01623	*ALDO*	*ALDO*; fructose-bisphosphate aldolase, class I [EC: 4.1.2.13]
	K03841	*FBP*	*FBP, fbp*; fructose-1,6-bisphosphatase I [EC: 3.1.3.11]
	K05298	*GAPA*	*GAPA*; glyceraldehyde-3-phosphate dehydrogenase (NADP+) (phosphorylating) [EC: 1.2.1.13]
	K01100	*At3g55800*	*E3.1.3.37*; sedoheptulose-bisphosphatase [EC: 3.1.3.37]
	K01803	*TPI*	*TPI, tpiA*; triosephosphate isomerase (TIM) [EC: 5.3.1.1]
	K00026	*MDH2*	*MDH2*; malate dehydrogenase [EC: 1.1.1.37]
	K01006	*ppdK*	*ppdK*; pyruvate, orthophosphate dikinase [EC: 2.7.9.1]
	K01595	*ppc*	*ppc*; phosphoenolpyruvate carboxylase [EC: 4.1.1.31]
	K14272	*GGAT*	*GGAT*; glutamate—glyoxylate aminotransferase [EC: 2.6.1.4 2.6.1.2 2.6.1.44]

**Table 8:** Compared with the control, the names of DEGs in the hormone-related metabolic pathways in cotton seedlings under mild and severe drought stress.

Pathway	K_ID	Gene	Gene Type
Carotenoid biosynthesis (ko00906)	K09840	*NCED*	*NCED*; 9-cis-epoxycarotenoid dioxygenase [EC: 1.13.11.51]
	K09841	*ABA2*	*ABA2*; xanthoxin dehydrogenase [EC: 1.1.1.288]
	K09842	*AAO3*	*AAO3*; abscisic-aldehyde oxidase [EC: 1.2.3.14]
Diterpenoid biosynthesis (ko00904)	K04125	*GA2ox*	*E1.14.11.13*; gibberellin 2beta-dioxygenase [EC: 1.14.11.13]
Tryptophan metabolism (ko00380)	K00128	*ALDH*	*ALDH*; aldehyde dehydrogenase (NAD+) [EC: 1.2.1.3]
	K01593	*DDC*	*DDC, TDC*; aromatic-L-amino-acid/L-tryptophan decarboxylase [EC: 4.1.1.28 4.1.1.105]
	K01426	*amiE*	*E3.5.1.4, amiE*; amidase [EC: 3.5.1.4]
	K11816	*YUCCA*	*YUCCA*; indole-3-pyruvate monooxygenase [EC: 1.14.13.168]
Signal transduction (ko04075, ko04016)	K14432	*ABF*	*ABF*; ABA responsive element binding factor
	K14496	*PYL*	*PYL*; abscisic acid receptor PYR/PYL family
	K14497	*PP2C*	*PP2C*; protein phosphatase 2C [EC: 3.1.3.16]
	K14498	*SNRK2*	*SNRK2*; serine/threonine-protein kinase SRK2 [EC: 2.7.11.1]
	K20716	*MAPKKK17/18*	*MAPKKK17_18*; mitogen-activated protein kinase kinase kinase 17/18
	K14494	*DELLA*	*DELLA*; DELLA protein
	K13946	*AUX1/LAX*	*AUX1, LAX*; auxin influx carrier (AUX1 LAX family)
	K14486	*ARF*	*K14486, ARF*; auxin response factor

**Table 9:** Primer information.

Gene Name	Primer Name	Primer Sequence (5′–3′)	Amplicon Size (bp)
*GA2OX1*	GA2OX1-F	TTGTTCCCTCCTCTTATCCC	233
	GA2OX1-R	GGTCCAATCCTTTTATTACCAT	233
*PSBO*	PSBO-F	TCGCTCTTGCTACATCTGC	115
	PSBO-R	CCTGTTCCTTTGACTTCCAT	115
*PSBP1*	PSBP1-F	CCACCACGCACTCACAAC	121
	PSBP1-R	TCCATCATCTTCCTGCTTTT	121
*PETE*	PETE-F	GGGGTCTGGCTTTCATTC	115
	PETE-R	CTTGGGATTTCGTCCTCAT	115
*PETH*	PETH-F	ATGCTCGCTACTGGAACTG	133
	PETH-R	GCAATGAACTACTCGTGGG	133
*RBCS*	RBCS-F	ACTACGATGGACGCTACTGG	108
	RBCS-R	CATTGGGGTATTCCTTCTTG	108
*FBA2*	FBA2-F	ACGGTCTTTCATCCCGCACAG	132
	FBA2-R	AGCGAGCAAGTCCCCAAGC	132
*GAPA*	GAPA-F	ACATCGTCCCGACTTCAA	168
	GAPA-R	CAGCGTTCACCTCTTCAGC	168
*TKL-2*	TKL-2-F	CCGTTTCATTCTGTCCGC	149
	TKL-2-R	CTCCAGGTGTTTCAAAGTTCTC	149
*UBC28*	UBC28-F	AGCGGATTTTGAAGGAACT	127
	UBC28-R	GCATAAGGGCTATCTGAGGG	127

**Table 10:** Reaction system.

Reagent	Volume (μL)	Step	Time	Cycles
2xqPCRmix	5.0	95 °C	30 s	
F Primer (10 pmol/μL)	0.25	95 °C	10 s	40 cycles
R Primer (10 pmol/μL)	0.25	60 °C	30 s	92.10%
DNA template	2.0	95 °C	15 s	
ddH_2_O	2.5	60 °C	60 s	fluorescence detection in 0.5 °C steps
total	10.0	95 °C	15 s	92.07%

## Data Availability

The data shown in this study are contained within the article. The raw data of the transcriptome has been uploaded to the NCBI under accession PRJNA1301586.
